# Supplementary material for: Biomimetic organo-hydrogels reveal the adipose tissue local mechanical anisotropy regulates ovarian cancer invasion
Source: Nat Commun. 2025 Sep 29;16:8541. doi: 10.1038/s41467-025-62296-7 (PMC12480658; doi:10.1038/s41467-025-62296-7)
Supplement: Supplementary file 1 — Supplementary Information [file 41467_2025_62296_MOESM1_ESM.pdf]

Supplementary Information

Biomimetic organo-hydrogels reveal the adipose tissue local mechanical anisotropy regulates ovarian cancer invasion

Jordi Gonzalez-Molina[1,2]*, Parisa Nabili[1,3], Daniele Marciano[2], Sara Abdelnabi[1], Okan Gultekin[1,3], Mohammed Fatih Rasul[3,4], Yikun Zhang[2], Clémence Nadal[2], Alexandra Chrysanthou[2], Twana Alkasalias[3,5], Sahar Salehi[3,6], Frances Balkwill[7], Kaisa Lehti[1,8], Julien E. Gautrot[2]*.

1. Department of Microbiology, Tumor and Cell Biology, Karolinska Institutet, Solnavägen 9, Solna 17165, Sweden.

2. School of Engineering and Materials Science, Queen Mary University of London, Mile End Road, London E1 4NS, United Kingdom.

3. Department of Women's and Children's Health, Division of Obstetrics and Gynecology, Karolinska Institutet, Solnavägen 9, Solna, Sweden.

4. Department of Pharmaceutical Basic Science, Faculty of Pharmacy, Tishk International University, Erbil, 44001, Iraq.

5. General Directorate of Scientific Research Centre, Salahaddin University-Erbil, Erbil, 44001, Iraq.

6. Department of Pelvic Cancer, Theme Cancer, Karolinska University Hospital, Stockholm, Sweden.

7. Barts Cancer Institute, Queen Mary University of London, Charterhouse Square, EC1M6BQ London, UK.

8. Department of Biomedical Laboratory Science, Norwegian University of Science and Technology - NTNU, Erling Skjalgssons gate 1, Trondheim 7491, Norway.

Correspondence: jordigonzalezmolina@gmail.com; j.gautrot@qmul.ac.uk

Supplementary Figures

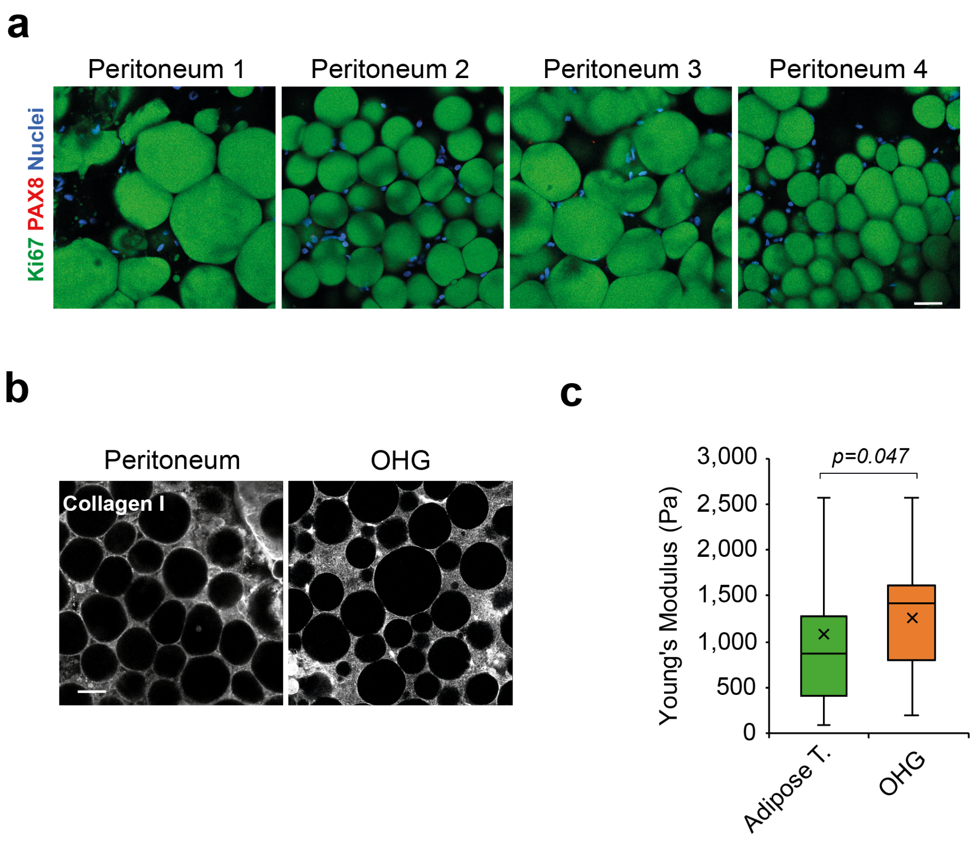

Supplementary Fig. 1: Comparison between human tissues and collagen-based OHG.

a, BODIPY (green), PAX8 (red), and nuclei (blue) staining of human peritoneal adipose tissues (n = 4 patients). **b**, Collagen-I (grey) staining of human peritoneal adipose tissue and OHG (n = 3 patients or gels). **c**, AFM quantification of the Young's modulus (n = 208 measurements from 3 patient tissues or OHGs). For the data in **c**, a two-sided unpaired t-test was performed. The boxplot shows the median (centre line), the interquartile range (IQR, box boundaries), and the whiskers extending to the minimum and maximum values. Scale bars, 50 μm (**a**, **b**). Source data are provided as a Source Data file.

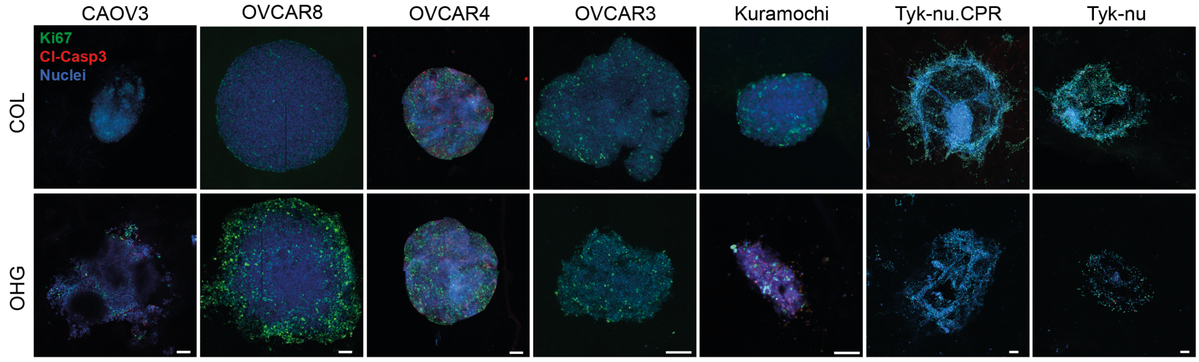

Supplementary Fig. 2: Ovarian cancer spheroid response to collagen and collagen-based OHGs.

Ki67 (green), cleaved-caspase 3 (red) and nuclei (blue) staining of spheroids embedded in collagen gels or OHGs after 7 d (representative images of n = 3 experiments). Scale bars, 100 μm.

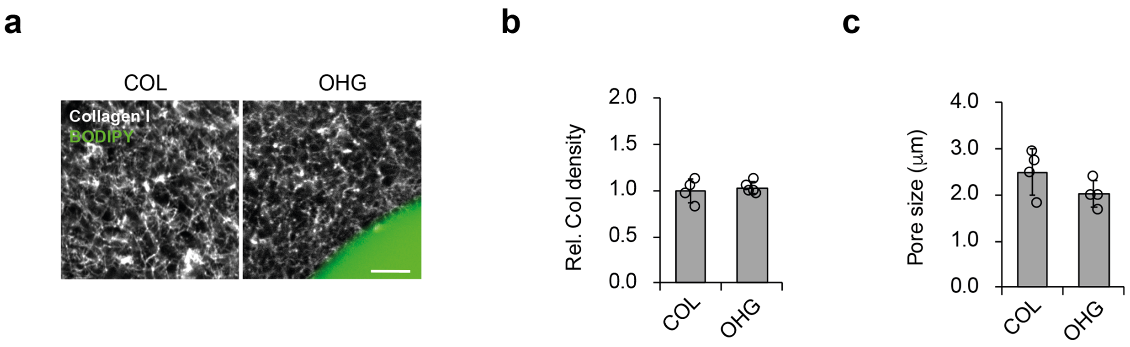

Supplementary Fig. 3: Collagen microstructure is comparable in collagen gels and OHGs.

a, Collagen-I (grey) and BODIPY (green) staining of a collagen gel and an OHG (representative images from n = 4 gels). **b**, Quantification of collagen density in collagen gels and OHG (n = 4 gels; average ± s.d.). **c**, Quantification of pore size in collagen gels and OHG (n = 4 gels; average ± s.d.). For the data in **b** and **c**, a two-sided unpaired t-test was performed. Scale bar, 10 μm (**a**). Source data are provided as a Source Data file.

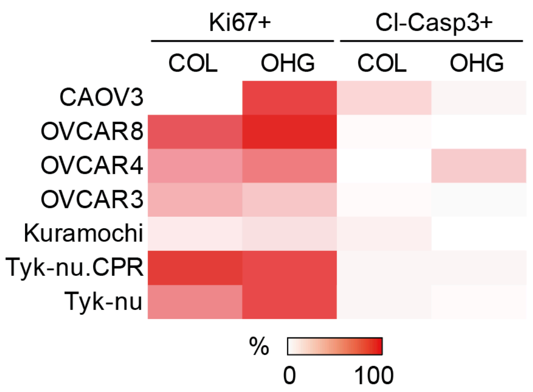

Supplementary Fig. 4: Ovarian Cancer cell proliferation and apoptosis in collagen and OHGs

Average percentage of Ki67+ and cleaved-caspase 3+ cells in collagen or OHG after 7 d in culture (n = 75 cells). Source data are provided as a Source Data file.

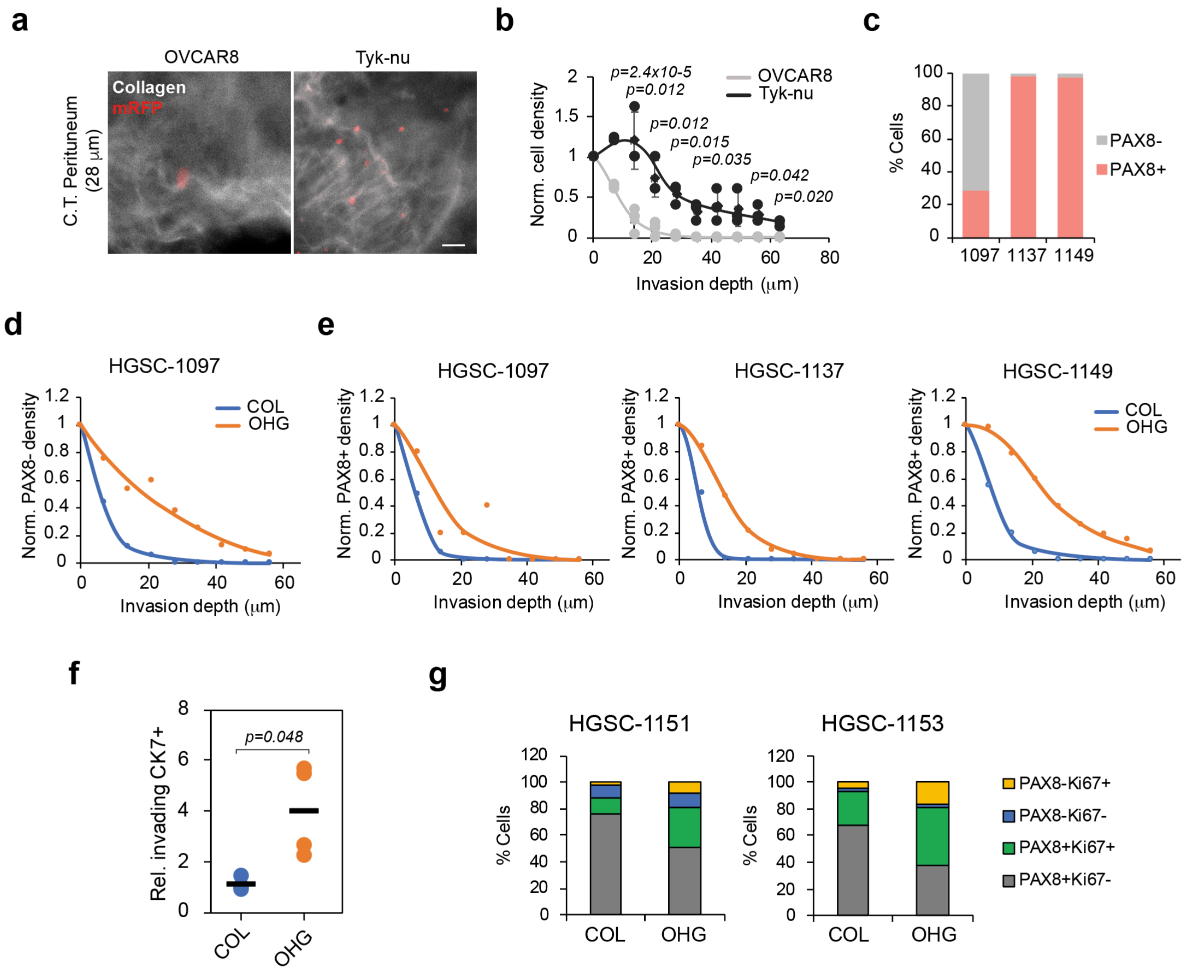

Supplementary Fig. 5: Invasion in OHG is enhanced compared to collagen gels.

a, Collagen-I (grey) and mRFP (red) staining of mRFP-OVCAR8 or mRFP-Tyk-nu cells 7 d after seeding on peritoneal connective tissues at 28 μm tissue depth (representative images from n = 3 tissues from 1 donor). **b**, Quantification of OVCAR8 and Tyk-nu organotypic invasion into peritoneal connective tissues after 7 d in culture (n = 3 tissues from 1 donor; average ± s.d.). **c**, Percentage of PAX8- and PAX8+ cells in malignant ascites after 7 d in culture (n ≥ 246 cells). **d**, Quantification of PAX8- cell organotypic hydrogel invasion after 7 d (n = 176 cells in 3 tissues from 1 donor). **e**, Quantification of PAX8+ cell organotypic hydrogel invasion after 7 d (n = 70 cells in 3 tissues from 3 donors). **f**, Quantification of invading single CK7+ cells relative to the number of spheroids from HGSC ascites cells embedded in collagen or OHG for 7 d (n = 4 gels from 1 donor). **g**, Quantification of Ki67+ cells in PAX8+ and PAX8- cells of HGSC ascites cells embedded in collagen or OHG for 7 d (n = 200 cells in 2 donors). For the data in **b** and **f** a two-sided unpaired t-test was performed. Scale bar, 50 μm (**a**). Source data are provided as a Source Data file.

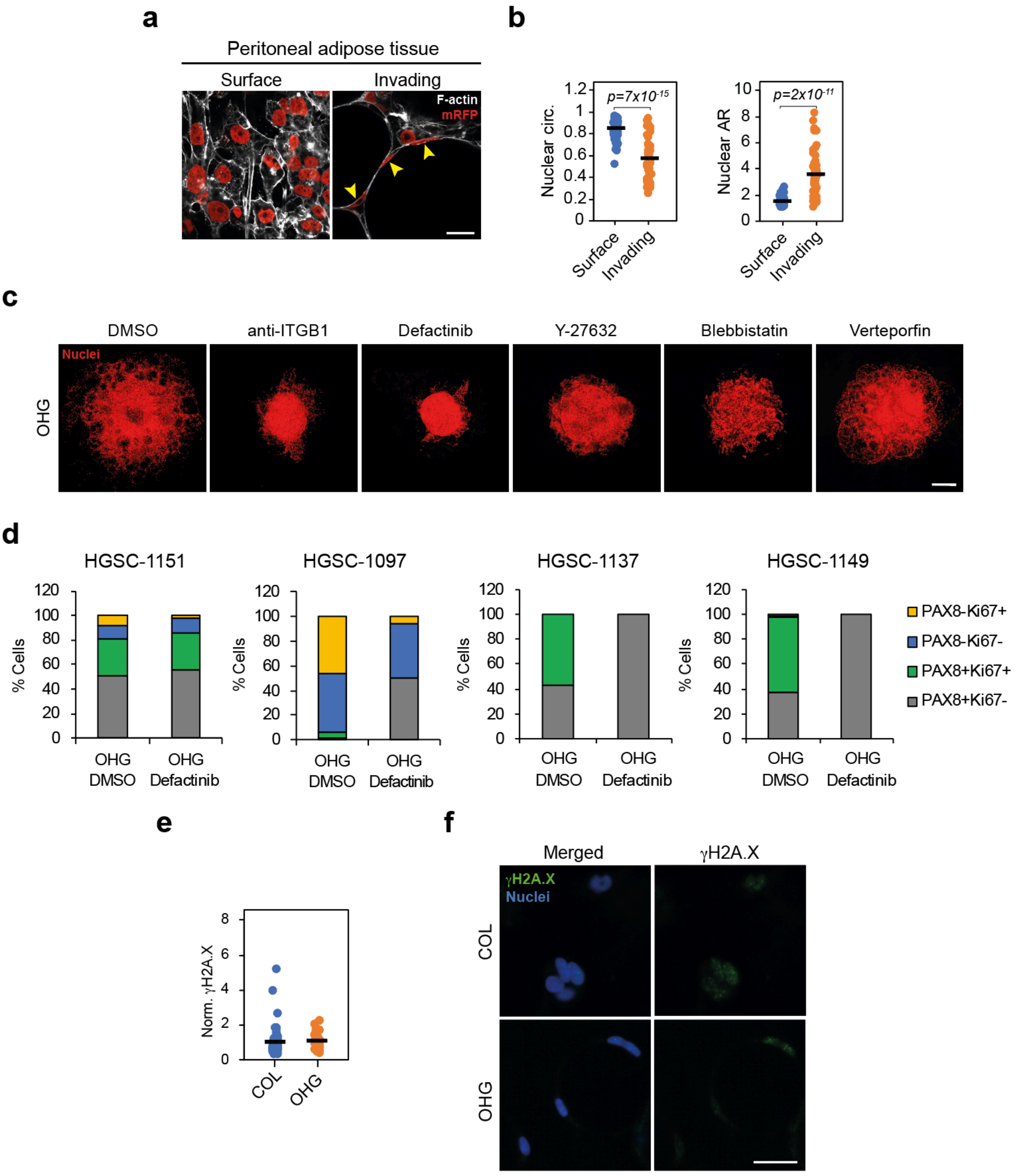

Supplementary Fig. 6: FAK-dependent cell invasion causes nuclear flattening but not DNA damage in ovarian cancer cells.

a, F-actin (grey) and mRFP (red) staining of mRFP-OVCAR8 cells invading human peritoneal adipose tissue explants (representative images from n = 4 tissues from one donor). Arrowheads indicate nuclei of mRFP-OVCAR8 migrating between adipocytes. **b**, Quantification of nuclear shape parameters in mRFP-OVCAR8 cells invading human peritoneal adipose tissue explants after 7 d in culture (n = 50 cells). **c**, Nuclei (red) staining of OVCAR8 spheroids embedded in OHGs for 7 d (representative images of n = 10 spheroids). **d**, Quantification of Ki67+ cells in PAX8+ and PAX8- cells of HGSC ascites cells embedded in collagen or OHG for 7 d (n = 43 cells in 4 donors).

e, Normalised intensity of γH2A.X immunofluorescence staining in OVCAR8 cells in collagen or OHG after 24 h in culture (n = 38 nuclei). **f**, γH2A.X (green) and nuclei (blue) staining of OVCAR8 cells in collagen or OHG after 24 h in culture (representative images from n = 3 gels). For the data in **b** and **e** a two-sided unpaired t-test was performed. Scale bars, 25 μm (**a**), 50 μm (**f**) and 200 μm (**c**). Source data are provided as a Source Data file.

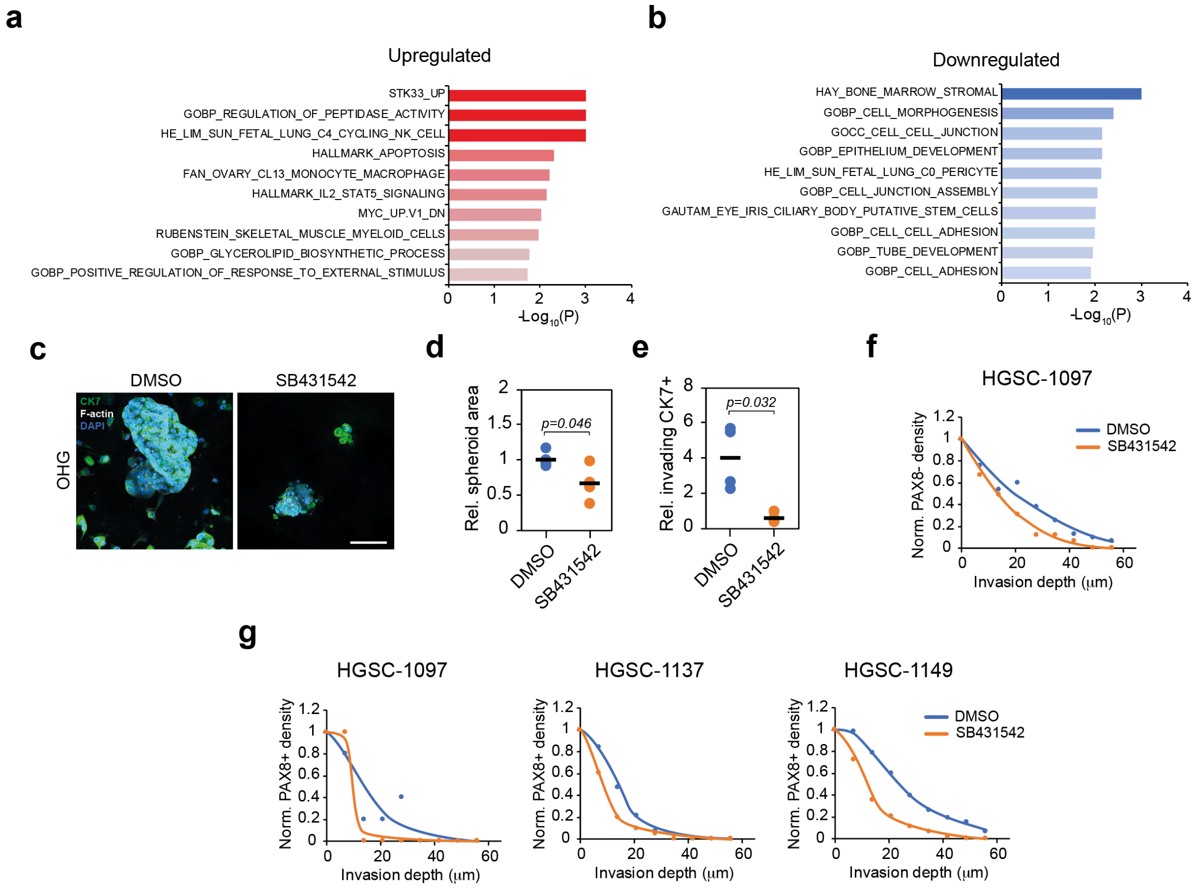

Supplementary Fig. 7: TGFβ signalling promotes invasion in OHG.

a, The ten most enriched gene sets from the most significantly upregulated genes in OHG-invasive ovarian cancer cell lines (Tyk-nu, OVCAR8, CAOV3) compared to non-invasive lines (OVCAR3, OVCAR4, Kuramochi). **b**, Ten most enriched gene sets from the significantly downregulated genes in OHG-invasive ovarian cancer cell lines compared to non-invasive lines. **c**, CK7 (green), F-actin (grey), and DAPI (blue) staining of HGSC ascites cells embedded in OHG for 7 d (representative images from n = 4 gels). **d**, Quantification of spheroid area of HGSC ascites cells embedded in OHG (n = 4 gels from 1 donor). **e**, Quantification of invading single CK7+ cells relative to the number of spheroids from HGSC ascites cells embedded in OHG (n = 4 gels from 1 donor). **f**, Quantification of PAX8- cell organotypic invasion depth after 7 d (n = 212 cells in 3 tissues from 1 donor). **g**, Quantification of PAX8+ cell organotypic invasion depth after 7 d (n = 125 cells in 3 tissues from 3 donors). For the data in **d** and **e** a two-sided unpaired t-test was performed. Scale bar, 100 μm (**c**). Source data are provided as a Source Data file.

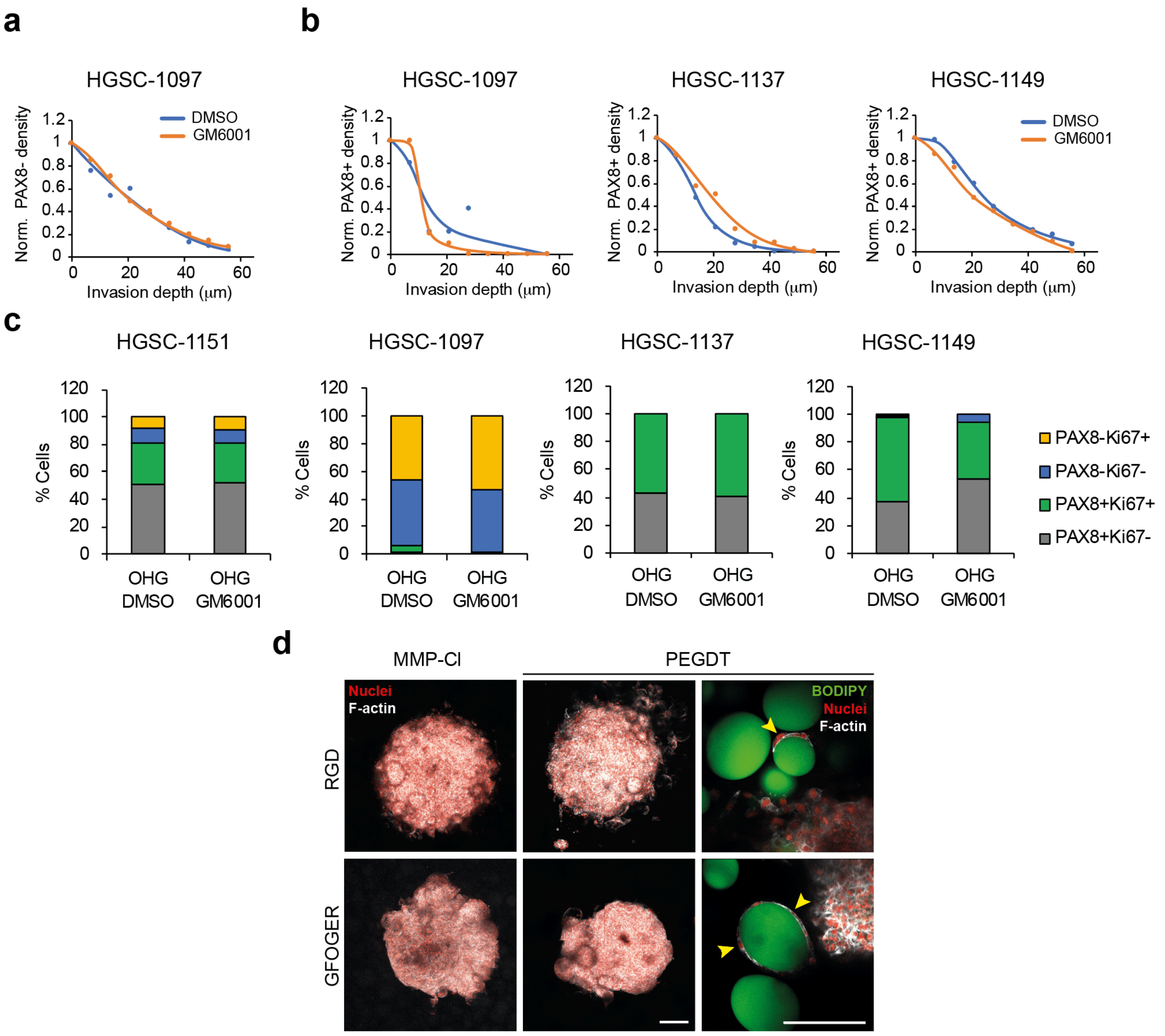

Supplementary Fig. 8: Cell invasion into adipose tissue is MMP-independent.

a, Quantification of PAX8- cell organotypic invasion depth after 7 days (n = 504 cells in 3 tissues from 1 donor). **b**, Quantification of PAX8+ cell organotypic invasion depth after 7 d (n = 38 cells in 3 tissues from 3 donors). **c**, Quantification of Ki67+ cells in PAX8+ and PAX8- cells of HGSC ascites cells embedded in OHG for 7 d (n = 54 cells in 4 donors). **d**, BODIPY (green), nuclei (red) and F-actin (grey) staining of OVCAR8 spheroids in MMP-Cleavable or non-cleavable (PEGDT) HA-NB OHG after 7 d in culture (representative images from n = 11 spheroids). Arrowheads indicate cells migrating at the microdroplet-ECM interface. Scale bars, 200 μm (**d**). Source data are provided as a Source Data file.

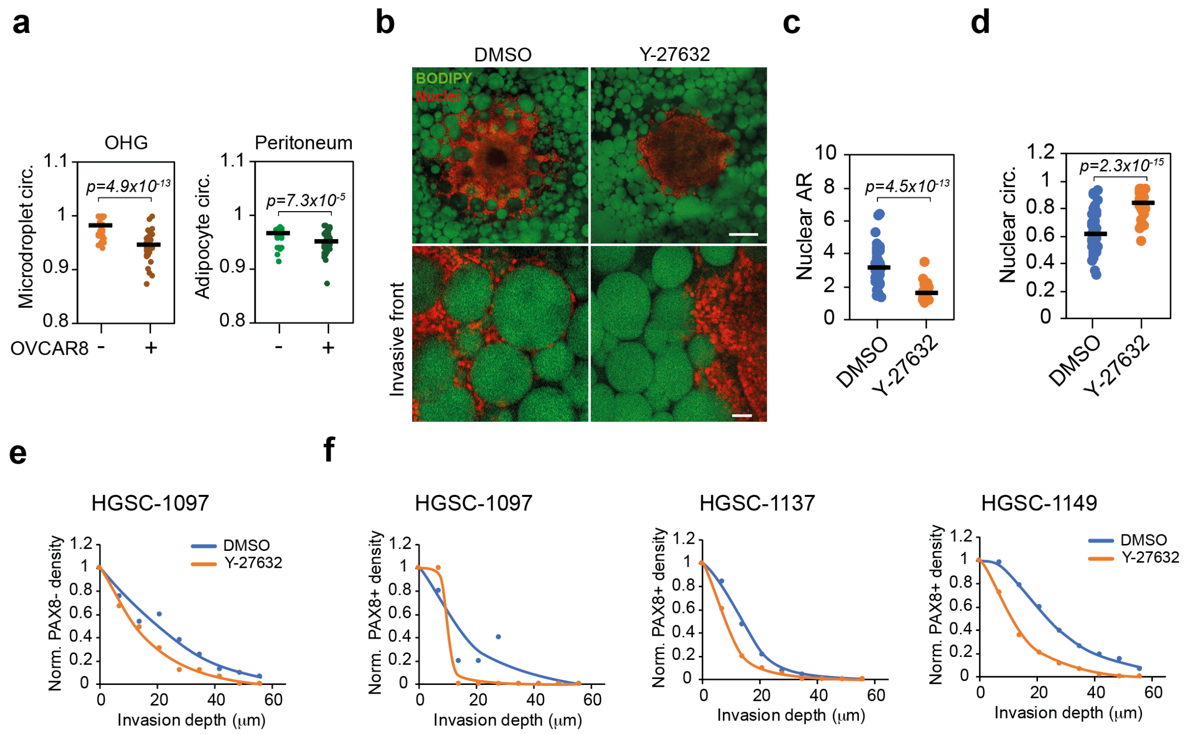

Supplementary Fig. 9: Cell invasion into adipose tissue is ROCK-dependent.

a, Quantification of the circularity of microdroplets and adipocytes in contact with OVCAR8 cells or OVCAR8 cell-free (n = 50). **b**, BODIPY (green) and nuclei (red) staining of OVCAR8 cells in collagen-based OHG after 7 d in culture (n = 10 spheroids). **c**, Quantification of nuclear aspect ratio of OVCAR8 cells in contact with microdroplets after 7 d culture (n = 50 cells). **d**, Quantification of nuclear circularity of OVCAR8 cells in contact with microdroplets after 7 d culture (n = 50 cells). **e**, Quantification of PAX8- cell organotypic invasion depth after 7 d (n = 158 cells in 3 tissues from 1 donor). **f**, Quantification of PAX8+ cell organotypic invasion depth after 7 d (n = 79 cells in 3 tissues from 3 donors). For the data in **a**, **c** and **d** a two-sided unpaired t-test was performed. Scale bars, 50 μm (bottom **b**), 200 μm (top **b**). Source data are provided as a Source Data file.

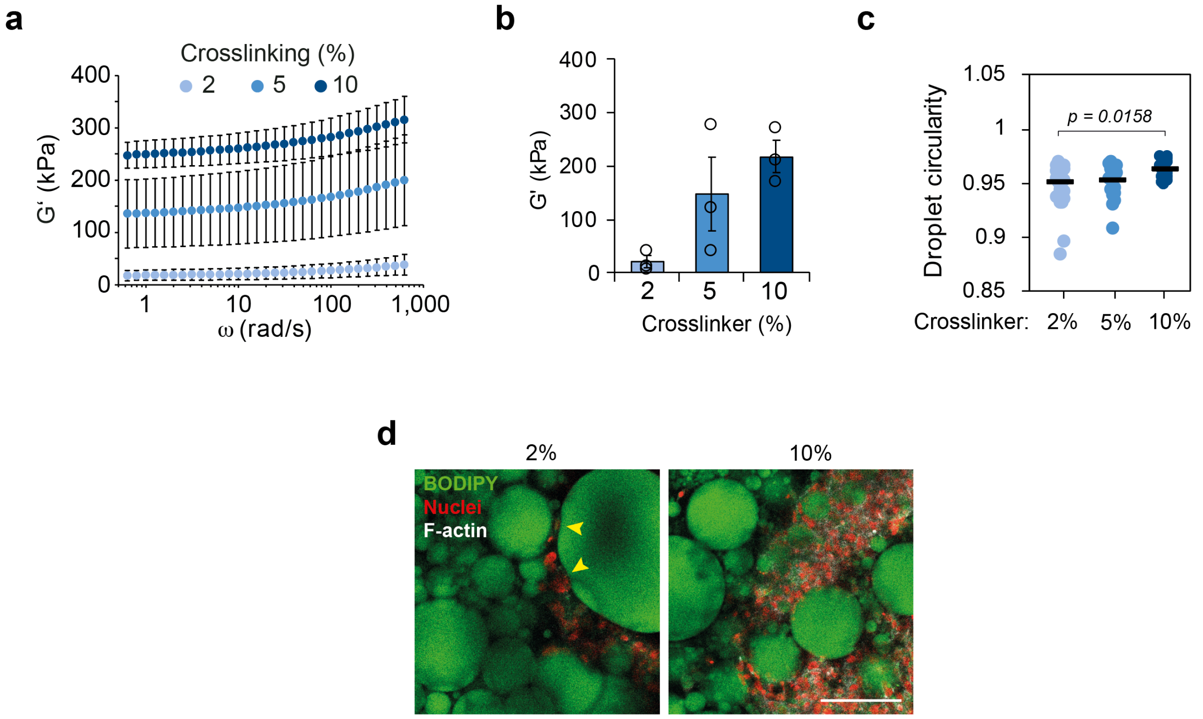

Supplementary Fig. 10: Cell-induced deformation is prevented in stiff PDMS microbeads.

a, **b**, Frequency sweeps (**a**) and shear storage moduli (at 10 rad/s) (**b**) of Sylgard 184 PDMS cured with increasing crosslinker concentrations (n = 3 PDMS gels; average ± s.d.). **c**, Quantification of the circularity of microbeads in contact with OVCAR8 cells after 7 d in culture (n = 25 microbeads). **d**, BODIPY (green), F-actin (grey), and nuclei (red) staining of OVCAR8 spheroids embedded for 7 d in PDMS OHGs crosslinked with collagen (representative images of n = 3 OHGs). Arrowheads indicate deformations caused by OVCAR8 cells. One-way analysis of variance (ANOVA) with Tukey's correction for multiple comparisons was performed for the data in **c**. Scale bar, 100 μm (**d**). Source data are provided as a Source Data file.

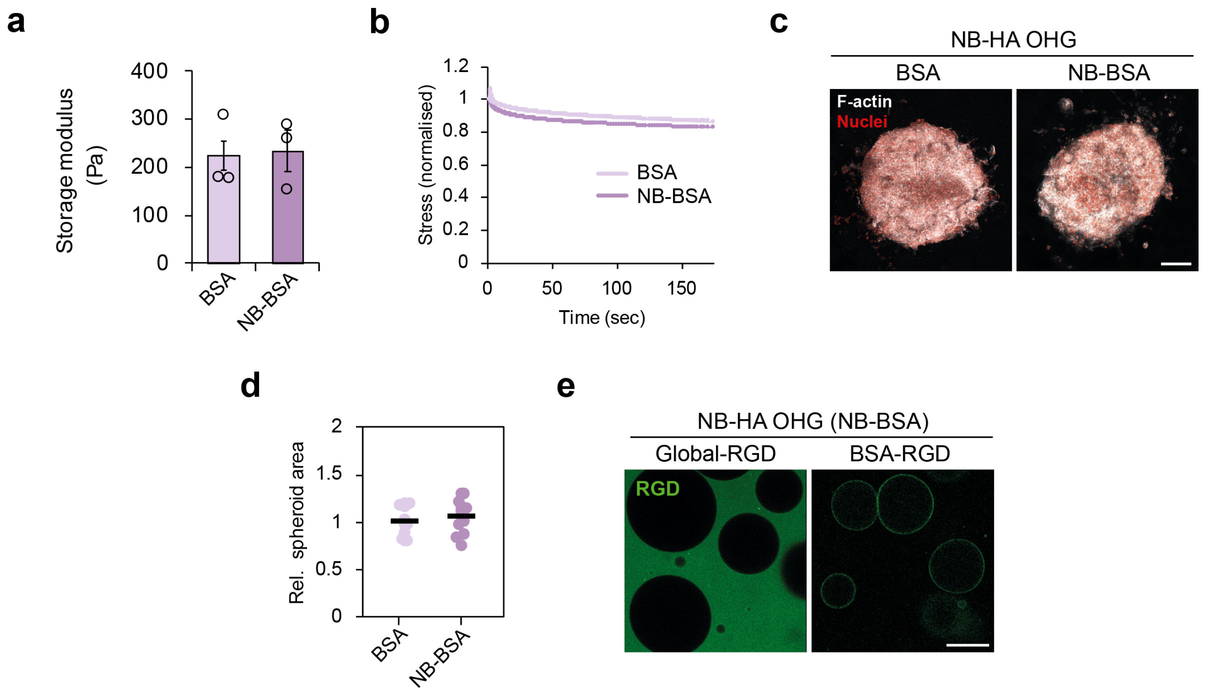

Supplementary Fig. 11: Mechanical characterisation, cell invasion, and RGD localisation in NB-HA OHGs.

a, Storage moduli of HA-NB OHGs formed with emulsions stabilised with BSA or NB-BSA nanosheets and crosslinked with PEGDT (n = 3 gels; average ± s.e.m.). **b**, Normalised stress-relaxation curves of HA-NB OHG formed with emulsions stabilised with BSA or NB-BSA nanosheets and crosslinked with PEGDT (n = 3 gels). **c**, F-actin (grey) and nuclei (red) staining of OVCAR8 spheroids after 7 d culture embedded in HA-NB OHG containing BSA or NB-BSA (crosslinked) microdroplets (representative images from n = 11 spheroids). **d**, Quantification of OVCAR8 spheroid area after culture in BSA or NB-BSA NB-HA OHG for 7 d, relative to BSA OHG average (n = 11 spheroids). **e**, RGD-FITC (green) in HA-NB OHG with controlled RGD localisation (representative images from n = 4 gels). For the data in **a** and **d** a two-sided unpaired t-test was performed. Scale bars, 50 μm (**e**), 200 μm (**c**). Source data are provided as a Source Data file.

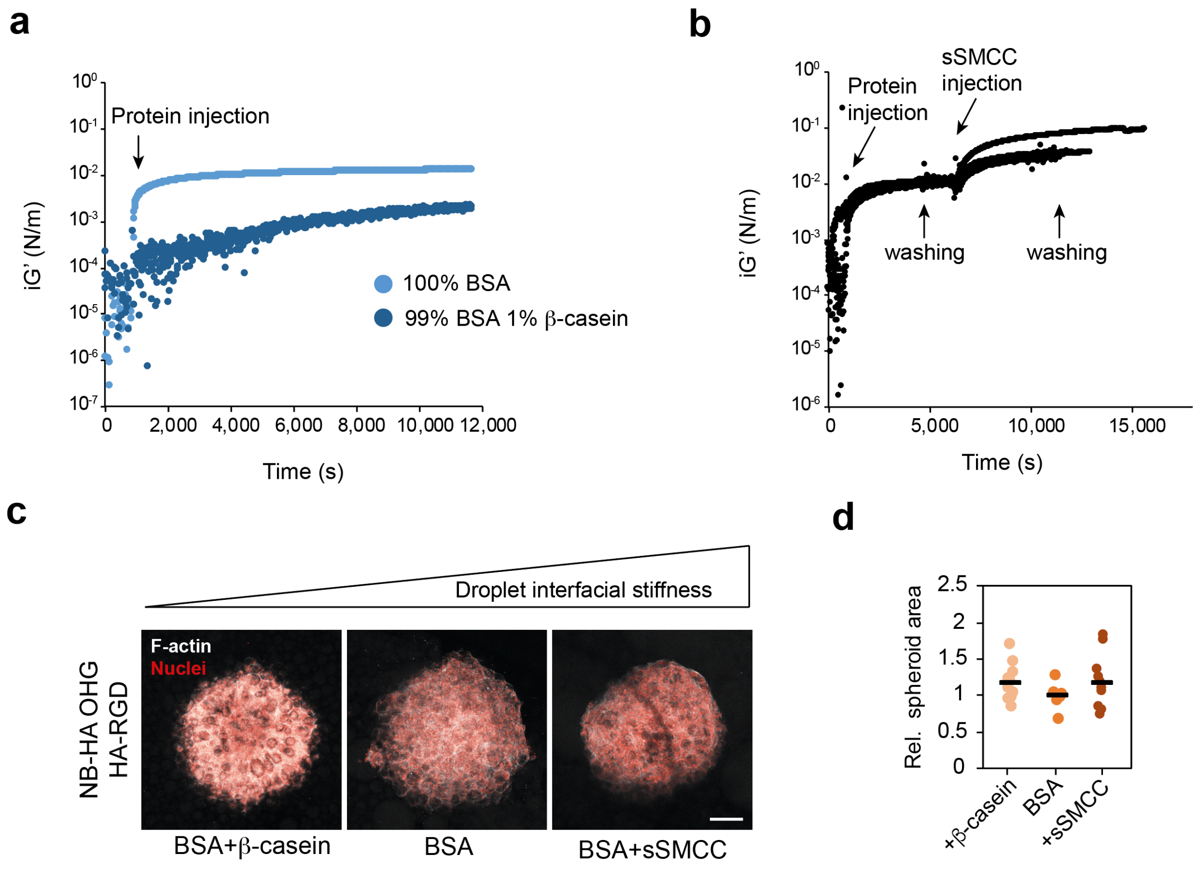

Supplementary Fig. 12: Interfacial protein nanosheet mechanics and its effect on cell invasion into OHGs.

a, Evolution of the interfacial shear storage modulus of 1 mg/ml BSA and BSA combined with β-casein protein nanosheets formed over NOVEC 7500 oil (0.1 Hz, 1.0x 10^{-4} rad). **b**, Evolution of the interfacial shear storage modulus of 1 mg/ml BSA before and after crosslinking with 2 mg/ml sulfo-SMCC. **c**, F-actin (grey) and nuclei (red) of OVCAR8 spheroids after 7 d in HA-NB OHGs with BSA microdroplet (i.e. RGD presented in the HA phase but not on the microdroplet surface) and varying protein nanosheet interfacial mechanics (representative images from n = 9 spheroids). **d**, OVCAR8 spheroid area in HA-NB OHGs with HA-presenting RGD and varying protein nanosheet interfacial mechanics after 7 d in culture, relative to BSA-only control (n = 9 spheroids). One-way analysis of variance (ANOVA) with Tukey's correction for multiple comparisons was performed for the data in **d**. Scale bar, 200 μm (**c**). Source data are provided as a Source Data file.

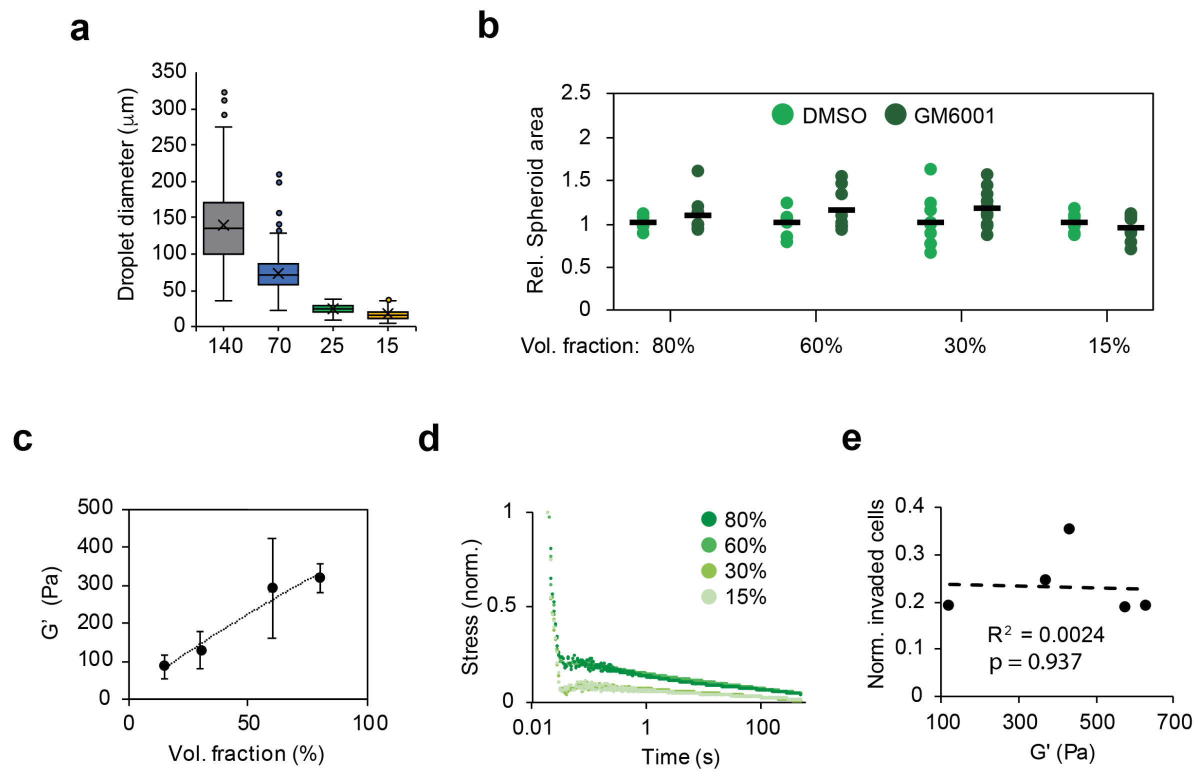

Supplementary Fig. 13: Impact of microdroplet size, volume fraction, and adipose tissue stiffness on cell invasion.

a, Quantification of microdroplet diameter of emulsions (n = 170 microdroplets). The boxplot shows the median (centre line), the interquartile range (IQR, box boundaries), and the whiskers extending 1.5x IQR, outliers shown separately. **b**, Quantification of OVCAR8 spheroid area relative to DMSO average after 7 d in 25 µm microdroplet OHGs. **c**, Storage modulus of 25 µm microdroplet OHGs with varying oil volume fraction. (n = 3 gels; average ± s.e.m.). Dashed line shows predicted trend. **d**, Normalised stress-relaxation curves of 25 µm microdroplet OHGs with varying oil volume fraction (n = 3 gels). **e**, Correlation between peritoneal tissue storage modulus and invaded OVCAR8 cells after 7 d at 42 µm depth (normalised to cell number at the tissue surface; n = 5 patients). For the data in **b** a two-sided unpaired t-test was performed. Coefficient of determination and Pearson correlation (two-tailed test) were performed in **e** to determine the relationship between tissue stiffness and OVCAR8 invasion. Dashed line shows predicted trend. Source data are provided as a Source Data file.

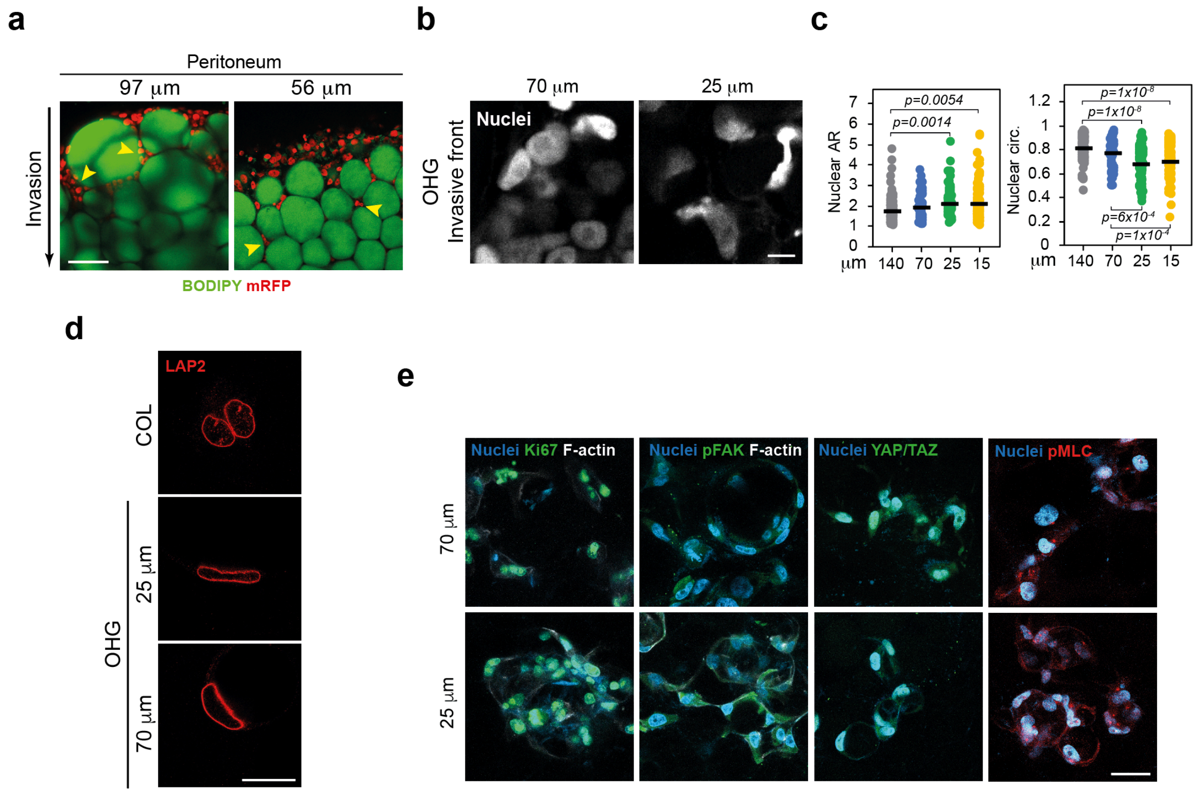

Supplementary Fig. 14: Microdroplet and adipocyte size regulate cell invasion.

a, BODIPY (green), mRFP (red) staining of mRFP-OVCAR8 cells invading into peritoneal adipose tissues after 7 d (representative images from n = 3 explants). Arrowheads indicate collectively-migrating cells (left) or single cell-migrating cells (right). Black arrow indicates the direction of invasion. **b**, Nuclei (grey) staining of OVCAR8 cells at the invasive front of spheroids embedded in OHG of distinct microdroplet size for 24 h (n = 3 gels). **c**, Quantification of nuclear shape descriptors of OVCAR8 cells from the invasive front of spheroids embedded in OHGs of varying microdroplet size for 24 h (n = 71 nuclei). **d**, LAP2 (red) staining of OVCAR8 cells embedded in collagen or in contact with microdroplets of OHGs with distinct microdroplet size for 24 h (n = 3 gels). **e**, Nuclei (blue), F-actin (grey), and (left to right) Ki67+ (green), pFAK (green), YAP/TAZ (green), and pMLC (red) staining of OVCAR8 cells embedded in OHG of 70 or 25 μm diameter microdroplets for 24 h (n = 3 gels). One-way analysis of variance (ANOVA) with Tukey's correction for multiple comparisons was performed for the data in **c**. Scale bars, 10 μm (**b**), 25 μm (**d**), 50 μm (**e**) and 100 μm (**a**). Source data are provided as a Source Data file.

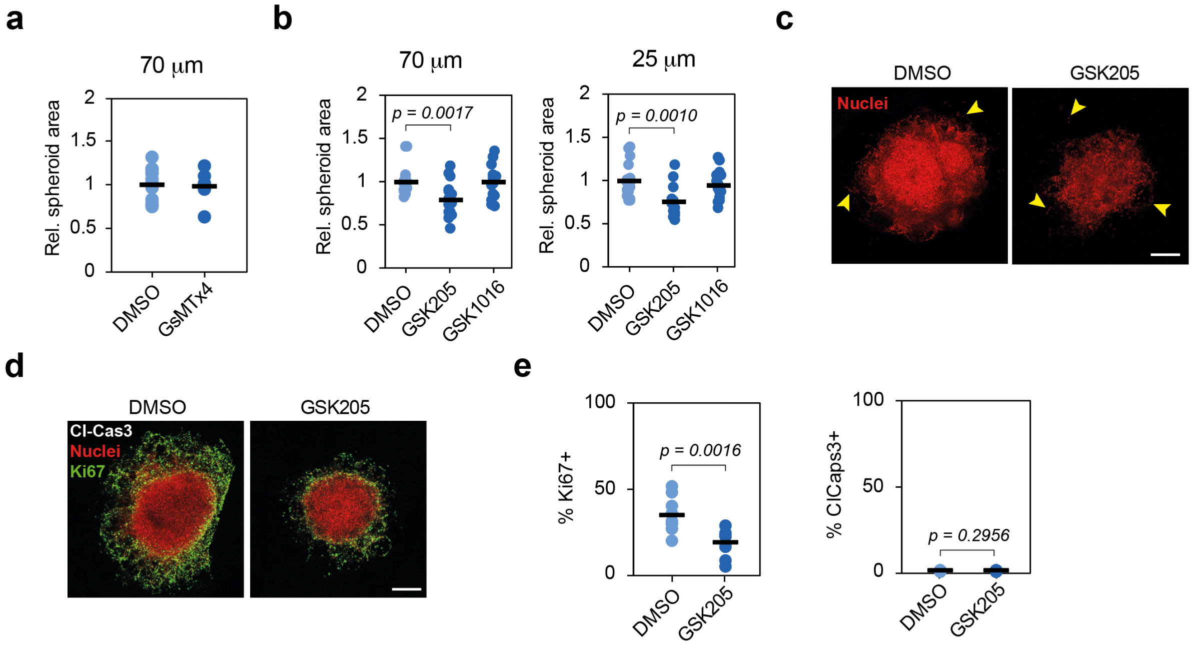

Supplementary Fig. 15: TRPV4 activity regulates cell proliferation.

a, Quantification of OVCAR8 spheroid area in OHG formed with 70 μm microdroplets upon inhibition with Piezo1 inhibitor GsMTx4 after 7 d in culture, relative to DMSO control average (n = 5 spheroids). **b**, OVCAR8 spheroid area in OHG formed with 70 or 25 μm microdroplets upon treatment with the TRPV4 inhibitor GSK205 or agonist GSK1016790A after 7 d in culture, relative to DMSO control average (n = 15 spheroids). **c**, Nuclei (red) of OVCAR8 cells embedded for 7 d in 70 μm OHGs (representative images from n = 15 spheroids). Arrowheads indicate cells at the invasive front. **d**, Cleaved Caspase 3 (grey), nuclei (red) and Ki67 (green) staining of OVCAR8 spheroids embedded in 70 μm microdroplet OHG for 7 d (representative images from n = 9 spheroids). **e**, Quantification of Ki67+ and Cleaved caspase 3+ cells from (**d**) (n = 9 spheroids). For the data in **a** and **e**, a two-sided unpaired t-test was performed. One-way analysis of variance (ANOVA) with Tukey's correction for multiple comparisons was performed for the data in **b**. Scale bar, 200 μm (**c** and **d**). Source data are provided as a Source Data file.

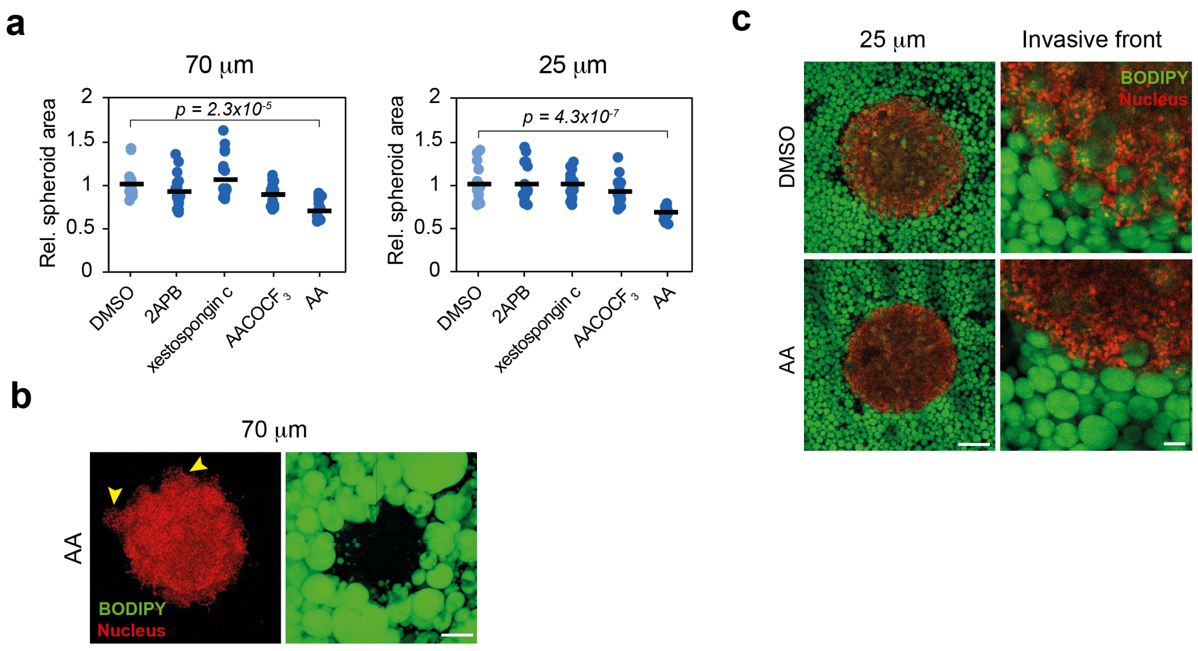

Supplementary Fig. 16: Inhibition of the cPLA2-Arachidonic acid-InsP3Rs pathway does not impair cell invasion.

a, Quantification of OVCAR8 spheroid area in OHG formed with 70 or 25 μm microdroplets with perturbed mechanical confinement signalling after 7 d in culture, relative to DMSO control (n = 16 spheroids). **b**, BODIPY (green) and nuclei (red) staining of OVCAR8 cells treated with arachidonic acid and embedded in 70 μm OHG for 7 d. Arrowheads indicate collectively-invading cells (representative images from n = 17 spheroids). **c**, BODIPY (green) and nuclei (red) of OVCAR8 cells embedded in 25 μm OHG for 7 d (representative images from n = 17 spheroids). One-way analysis of variance (ANOVA) with Tukey's correction for multiple comparisons was performed for the data in **a**. Scale bars, 200 μm (**b**, left **c**), 25 μm (right **c**). Source data are provided as a Source Data file.

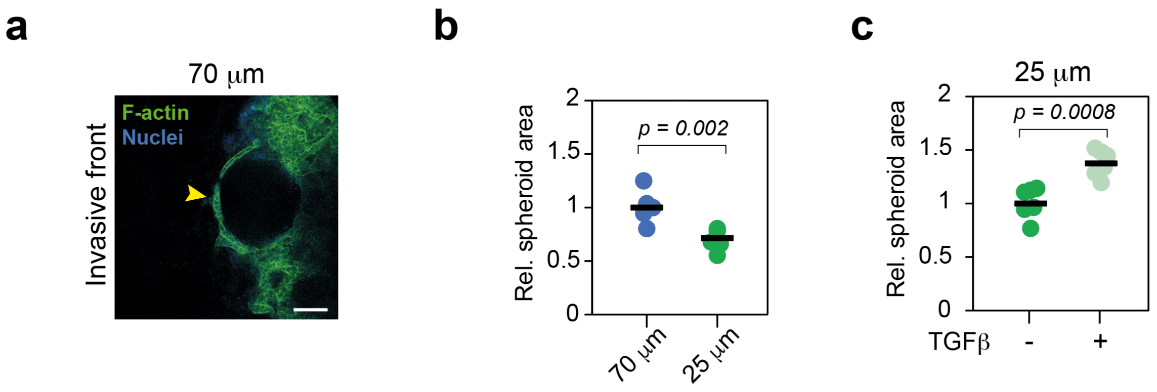

Supplementary Fig. 17: CAOV3 invasion is regulated by microdroplet size.

a, F-actin (green) and nuclei (blue) staining of CAOV3 cells invading into a 70 μm OHG. Arrowhead indicates collectively-migrating CAOV3 cells at the invasive front. **b**, Quantification of CAOV3 spheroid area relative to 70 μm OHG average after 7 d (n = 6 spheroids). **c**, Quantification of CAOV3 spheroid area relative to TGF-β-free control after 7 d in 25 μm OHGs (n = 6 spheroids). For the data in **b** and **c**, a two-sided unpaired t-test was performed. Scale bars, 100 μm (**a**). Source data are provided as a Source Data file.
