# Supplementary material for: Biomimetic organo-hydrogels reveal the adipose tissue local mechanical anisotropy regulates ovarian cancer invasion
Source: Nat Commun. 2025 Sep 29;16:8541. doi: 10.1038/s41467-025-62296-7 (PMC12480658; doi:10.1038/s41467-025-62296-7)
Supplement: Supplementary file 2 — Reporting Summary [file 41467_2025_62296_MOESM2_ESM.pdf]

nature portfolio

Corresponding author(s): Jordi Gonzalez-Molina and Julien Gautrot

Last updated by author(s): Jul 7, 2025

Reporting Summary

Nature Portfolio wishes to improve the reproducibility of the work that we publish. This form provides structure for consistency and transparency in reporting. For further information on Nature Portfolio policies, see our Editorial Policies and the Editorial Policy Checklist.

Statistics

For all statistical analyses, confirm that the following items are present in the figure legend, table legend, main text, or Methods section.

n/a	Confirmed	
☐	☒	The exact sample size (n) for each experimental group/condition, given as a discrete number and unit of measurement
☐	☒	A statement on whether measurements were taken from distinct samples or whether the same sample was measured repeatedly
☐	☒	The statistical test(s) used AND whether they are one- or two-sided<br>*Only common tests should be described solely by name; describe more complex techniques in the Methods section.*
☐	☒	A description of all covariates tested
☐	☒	A description of any assumptions or corrections, such as tests of normality and adjustment for multiple comparisons
☐	☒	A full description of the statistical parameters including central tendency (e.g. means) or other basic estimates (e.g. regression coefficient) AND variation (e.g. standard deviation) or associated estimates of uncertainty (e.g. confidence intervals)
☐	☒	For null hypothesis testing, the test statistic (e.g. F, t, r) with confidence intervals, effect sizes, degrees of freedom and P value noted<br>*Give P values as exact values whenever suitable.*
☒	☐	For Bayesian analysis, information on the choice of priors and Markov chain Monte Carlo settings
☒	☐	For hierarchical and complex designs, identification of the appropriate level for tests and full reporting of outcomes
☐	☒	Estimates of effect sizes (e.g. Cohen's d, Pearson's r), indicating how they were calculated

Our web collection on statistics for biologists contains articles on many of the points above.

Software and code

Policy information about availability of computer code

Data collection	Confocal microscopy: Zeiss LSM 800 Airy or a Zeiss LSM 900 Airy laser-scanning confocal microscope operated by the ZEN Blue v2.6 software; rheology: hybrid rheometer (DHR-3) from TA Instruments and TRIOS software v5.8 (for interfacial rheology: using a Du Nouy ring geometry and a Delrin trough with a circular channel); atomic force microscopy: JPK NanoWizard 4 (JPK Instruments) with SPM software (JPK Instruments).
Data analysis	Gene expression analysis: Affymetrix Expression Console Software (version 4.0.1; Thermofisher) and Gene Set Enrichment Analysis (GSEA; v 4.3.2). Image analyses: ImageJ (Fiji) (v 2.14.0/1.54f). Statistical analyses: Microsoft excel (v 16.16.27) and GraphPad Prism (v 10.4.1). AFM analysis: Matlab (v9.14) using a classic Hertz model for a spherical tip.

For manuscripts utilizing custom algorithms or software that are central to the research but not yet described in published literature, software must be made available to editors and reviewers. We strongly encourage code deposition in a community repository (e.g. GitHub). See the Nature Portfolio guidelines for submitting code & software for further information.

Data

Policy information about availability of data

All manuscripts must include a data availability statement. This statement should provide the following information, where applicable:

- Accession codes, unique identifiers, or web links for publicly available datasets
- A description of any restrictions on data availability
- For clinical datasets or third party data, please ensure that the statement adheres to our policy

The data generated in this study are provided in the Supplementary figures and Source Data file. The gene expression data of human cell lines used in this study are available in the Gene Expression Omnibus (GEO) repository under the accession codes GSE50831 and GSE28724.

Research involving human participants, their data, or biological material

Policy information about studies with human participants or human data. See also policy information about sex, gender (identity/presentation), and sexual orientation and race, ethnicity and racism.

Reporting on sex and gender	Sex was determined based on medical records and clinical diagnosis of ovarian cancer, which inherently implies assignment as female at birth. Gender identity was not collected, and no gender-based analyses were conducted.
Reporting on race, ethnicity, or other socially relevant groupings	This study did not involve human participants but used human biological material. Race, ethnicity, or other socially relevant groupings were not considered.
Population characteristics	Patient age ranged between 48 – 78 years and body mass index ranged between 19 – 36. Patients were diagnosed with High Grade Serous Ovarian Cancer and had not receive neoadjuvant chemotherapy.
Recruitment	The samples used in this study contain biobanked and fresh material from female HGSC patients who have been submitted to surgery at the Karolinska Institute Hospital, Sweden.
Ethics oversight	All participants provided written informed consent, and the study was approved by the Swedish Ethical Review Authority (Dnr: 2019-05149 and Dnr 2023-04696-01). All procedures were performed in accordance with relevant guidelines and regulations and adhered to the principles of the Declaration of Helsinki.

Note that full information on the approval of the study protocol must also be provided in the manuscript.

Field-specific reporting

Please select the one below that is the best fit for your research. If you are not sure, read the appropriate sections before making your selection.

☒ Life sciences ☐ Behavioural & social sciences ☐ Ecological, evolutionary & environmental sciences

For a reference copy of the document with all sections, see nature.com/documents/nr-reporting-summary-flat.pdf

Life sciences study design

All studies must disclose on these points even when the disclosure is negative.

Sample size	Data from patient-derived tissues was collected from at least five donors, and patient-derived cells were obtained from at least 3 patients. All other experiments were conducted at least in triplicates. No statistical analyses were used to predetermine the sample size. Sample size was estimated based on common practice in the field.
Data exclusions	No data was excluded.
Replication	Replication of data was carried out in independent separate experiments. We repeated experiments performed in organo-hydeogel models using human adipose tissues to confirm our findings. All attempts at replication were successful.
Randomization	Experiments were not randomized, as is common practice in the research field.
Blinding	Blinding was not used for data collection or analysis, as is common practice in the research field.

Reporting for specific materials, systems and methods

We require information from authors about some types of materials, experimental systems and methods used in many studies. Here, indicate whether each material, system or method listed is relevant to your study. If you are not sure if a list item applies to your research, read the appropriate section before selecting a response.

Materials & experimental systems

n/a	Involved in the study	
☐	☒	Antibodies
☐	☒	Eukaryotic cell lines
☒	☐	Palaeontology and archaeology
☒	☐	Animals and other organisms
☒	☐	Clinical data
☒	☐	Dual use research of concern
☒	☐	Plants

Methods

n/a	Involved in the study	
☒	☐	ChIP-seq
☒	☐	Flow cytometry
☒	☐	MRI-based neuroimaging

Antibodies

Antibodies used

Primary antibodies: Ki67 (1:400, 9449S, Lot. 12, Cell Signaling Technology) and cleaved Caspase-3 (1:800, 9664, Lot. 22, Cell Signaling Technology), Collagen-I (1:800, MA1-26771, Lot. XA3474955, Thermo Fisher Scientific), pFAK (1:200, phosphorylated (Y397) FAK, 611807, Lot. 6092601, BD Biosciences), pMLC (1:400, phosphorylated (S20) myosin light chain, ab2480, Lot. GR3241064-7, Abcam), YAP/TAZ (1:200, sc-101199, Lot. J3013, Santa Cruz Biotechnology),H2A.X (1:200, phosphorylated (S139) gamma H2AX, #2577S, Lot.11, Cell Signaling Technologies), LAP2 (1:400, PA5-52519, Thermo Fisher Scientific), PAX8 (1:400, 10336-1-AP, Lot. 00099975, Thermo Fisher Scientific), CK7 (1:400, MA5-11986, Thermo Fisher Scientific). Secondary antibodies: Alexa Fluor Plus 488 anti-mouse IgG (1:1000, A-21202, Lot. VC300588, Thermo Fisher), Alexa Fluor 350 anti-rabbit IgG (1:500, A10039, Lot. 2533800, Thermo Fisher) and Alexa Fluor Plus 555 anti-rabbit IgG (1:1000, A32794, Lot. VI311611, Thermo Fisher), Alexa Fluor 647 anti-rabbit IgG (1:500, A31573, Lot. 1826679, Thermo Fisher Scientific), Alexa Fluor 647 anti-mouse IgG (1:500, A31571, Lot. 1900251, Thermo Fisher Scientific).

Validation

The subcellular localization of the primary antibodies used for immunofluorescence was compared with the examples or references provided by the manufacturers. Websites of the manufacturers:
Ki67 (9449S, Cell Signaling Technology): https://www.cellsignal.com/products/primary-antibodies/ki-67-8d5-mouse-mab/9449?srsltid=AfmBOorvJZYloJx2795cNRQ8S67AKyzsSEvO405BypUDSI-mED0PfX2I
Cleaved Caspase-3 (9664, Cell Signaling Technology): https://www.cellsignal.com/products/primary-antibodies/cleaved-caspase-3-asp175-5a1e-rabbit-mab/9664
Collagen-I (MA1-26771, Thermo Fisher Scientific): https://www.thermofisher.com/antibody/product/Collagen-I-Antibody-clone-COL-1-Monoclonal/MA1-26771
pFAK (phosphorylated (Y397) FAK, BD Biosciences): https://www.bdbiosciences.com/en-at/products/reagents/microscopy-imaging-reagents/immunofluorescence-reagents/purified-mouse-anti-human-fak-py397.611807
pMLC (phosphorylated (S20) myosin light chain, ab2480, Abcam): https://www.abcam.com/en-us/products/primary-antibodies/myl12a-phospho-s19-antibody-ab2480?srsltid=AfmBOoo6xU1FbmcviolRDJi5P3z32HP5yxBhGM9ErrpWdF5_4pc-qq9s
YAP/TAZ (sc-101199, Santa Cruz Biotechnology): https://www.scbt.com/es/p/yap-antibody-63-7?srsltid=AfmBOoothh2_FBi6PWBbgcU9zz6VxyU-hXGfHVXhV2Zte8MKd_pz9-Q4
gH2A.X (phosphorylated (S139) gamma H2AX, Cell Signaling Technologies): https://www.cellsignal.com/products/primary-antibodies/phospho-histone-h2a-x-ser139-antibody/2577?srsltid=AfmBOoqrXON4Dx6deYQ1Z2lTAJ0FxuBF0UvJBIBHnOmvN6O6_Q2xCbMy
LAP2 (PA5-52519, Thermo Fisher Scientific): https://www.thermofisher.com/antibody/product/LAP2-Antibody-Polyclonal/PA5-52519
PAX8 (10336-1-AP, Thermo Fisher Scientific): https://www.thermofisher.com/antibody/product/PAX8-Antibody-Polyclonal/10336-1-AP
CK7 (MA5-11986, Thermo Fisher Scientific): https://www.thermofisher.com/antibody/product/Cytokeratin-7-Antibody-clone-OV-TL-12-30-Monoclonal/MA5-11986
Alexa Fluor Plus 488 anti-mouse IgG (A-21202, Thermo Fisher): https://www.thermofisher.com/antibody/product/Donkey-anti-Mouse-IgG-H-L-Highly-Cross-Adsorbed-Secondary-Antibody-Polyclonal/A-21202
Alexa Fluor 350 anti-rabbit IgG (A10035, Thermo Fisher): https://www.thermofisher.com/antibody/product/Donkey-anti-Mouse-IgG-H-L-Highly-Cross-Adsorbed-Secondary-Antibody-Polyclonal/A10035
Alexa Fluor Plus 555 anti-rabbit IgG (A32794, Thermo Fisher): https://www.thermofisher.com/antibody/product/Donkey-anti-Rabbit-IgG-H-L-Highly-Cross-Adsorbed-Secondary-Antibody-Polyclonal/A32794
Alexa Fluor 647 anti-rabbit IgG (A31573, Thermo Fisher Scientific): https://www.thermofisher.com/antibody/product/Donkey-anti-Rabbit-IgG-H-L-Highly-Cross-Adsorbed-Secondary-Antibody-Polyclonal/A-31573
Alexa Fluor 647 anti-mouse IgG (A31571, Thermo Fisher Scientific): https://www.thermofisher.com/antibody/product/Donkey-anti-Mouse-IgG-H-L-Highly-Cross-Adsorbed-Secondary-Antibody-Polyclonal/A-31571

Eukaryotic cell lines

Policy information about cell lines and Sex and Gender in Research

Cell line source(s)

OVCAR8 (305383, Cytion), OVCAR4 (SCC258, Sigma), Kuramochi (JCRB0098, Japanese Collection of Research Bioresources Cell Bank), CAOV3 (HTB-75, ATCC), Tyk-nu (JCRB0234.0, Japanese Collection of Research Bioresources Cell Bank), and Tyk-nu.CPR (JCRB0234.1, Japanese Collection of Research Bioresources Cell Bank).

Authentication

None of the cell lines were further authenticated.

Mycoplasma contamination

Cell lines in this study were routinely tested negative for mycoplasma.

Commonly misidentified lines (See ICLAC register)	None of the cell lines used have been listed as commonly misidentified lines by the ICLAC.

Plants

Seed stocks	N/A
Novel plant genotypes	N/A
Authentication	N/A
